# Experiences of youth and caregivers waiting for mental health services in the UK: a qualitative study to inform policy and practice

**DOI:** 10.1007/s00787-025-02952-x

**Published:** 2026-01-05

**Authors:** Emeline Han, Alexandra Burton, Alexandra Bradbury, Daniel Hayes, Joely Wright, Lou Sticpewich, Joanna Page, Daisy Fancourt

**Affiliations:** Social Biobehavioural Research Group, Research Department of Behavioural Science and Health, Institute of Epidemiology & Health Care, University College London, 1-19 Torrington, London, WC1E 7HB UK

**Keywords:** Child and adolescent mental health services, Mental health policy, Qualitative research, Lived experiences, Coping strategies

## Abstract

Long waitlists are the most commonly reported barrier to accessing mental health services in the UK and across Europe. Yet, we have almost no understanding of the lived experiences of waiting among youth and their caregivers. In this qualitative study, we conducted semi-structured interviews with a purposive sample of 20 youth (aged 11–17) and 15 caregivers from ten child and adolescent mental health services (CAMHS) sites geographically spread across England. We used reflexive thematic analysis to analyse the data. We generated four themes that characterised participants’ experiences of waiting: (1) decline in mental and physical health, (2) strain on family dynamics and wider relationships, (3) unclear processes and communication, and (4) perceived mismatch between need and support. We also generated four themes illustrating participants’ coping strategies while waiting: (1) using self-help and parenting resources, (2) engaging in hobbies, (3) relying on social support, and (4) seeking alternative services. There is an urgent need to shorten CAMHS wait times as our findings show the adverse impact of waiting on youth and their families, with mental health worsening not just due to time passing but as a direct result of being put on a waitlist. While youth on CAMHS waitlists make active efforts to manage their symptoms, limitations to these coping strategies suggest that improved information sharing and tailored interim support is needed to mitigate against mental health deterioration while waiting.

**Supplementary Information:**

The online version contains supplementary material available at 10.1007/s00787-025-02952-x.

## Introduction

Despite growing recognition of the importance of early intervention [1], long waits for mental health services have become “normalised”, according to a recent review of the National Health Service (NHS) in England by Lord Darzi [2]. As of September 2024, 352,682 youth under 18 were waiting for child and adolescent mental health services (CAMHS), with a median wait time of almost eight months and 90th percentile wait time of more than two years [3]. These national averages far exceed the government’s proposed target of a four-week waiting time standard for CAMHS by 2022/23 [4]. Notably, these reported wait times reflect the period from referral to first contact, such as an initial assessment, and not the full duration between referral to treatment. In the UK, referrals to CAMHS are most often made by general practitioners (GPs), school staff, or social care professionals, which are then reviewed and triaged by multidisciplinary CAMHS teams [5]. Majority of cases accepted onto CAMHS waitlists for specialist treatment are youth presenting moderate to severe difficulties, while those falling below thresholds are typically signposted to lower-intensity services [5].

CAMHS waiting times vary across the UK, with longer waits more common in socioeconomically deprived areas due to higher rates of mental health problems and lower levels of service provision [6]. Prolonged waits for mental health services are associated with heightened psychological distress, increased maladaptive coping strategies, reduced engagement with treatment, and poorer treatment outcomes [7–9]. In a 2022 survey of 14,000 youth in the UK, 58% felt their mental health deteriorated and 26% attempted suicide while waiting for support [10]. In the absence of timely intervention, some youth present to Accident and Emergency (A&E) services (i.e., hospital emergency departments) in crisis, adding strain on services that are not designed to manage ongoing psychological needs [11].

While quantitative studies have documented alarming statistics on wait times and their consequences, there remains limited qualitative research on the lived experiences of waiting for mental health care. Existing literature has primarily explored broader experiences of accessing services, with complex referral pathways, long waitlists, and fragmented care commonly reported as barriers to access in the UK and across Europe [12, 13]. Few studies have specifically examined experiences of being on a mental health waitlist. In one study conducted almost 20 years ago, parents of children on CAMHS waitlists described feelings of loneliness, abandonment, and self-blame [14]. In more recent research, young adults waiting for mental health services reported negative emotions including anger, frustration, and hopelessness, as well as a decline in both mental and physical functioning [15, 16]. These adults developed a range of coping mechanisms while waiting, some of which were adaptive (e.g., mindfulness, meditation), while others were maladaptive (e.g., self-harm, substance abuse) [15, 16].

To date, no qualitative study has focused on children’s and adolescents’ experiences of waiting for mental health services. The European Society for Child and Adolescent Psychiatry (ESCAP) has recently called for urgent action from Member States to address the growing youth mental health crisis, and highlighted the importance of actively involving youth with lived experience in these efforts [17]. Therefore, the aim of this qualitative study was to examine the experiences and coping strategies of youth on the CAMHS waitlist, looking across adolescence (ages 11–18), triangulating youth and caregiver perspectives, and using purposive sampling approaches. In this context, coping refers to how youth managed their mental health symptoms and supported their wellbeing while waiting for formal services. The findings have immediate and urgent relevance to policy development in the UK and Europe.

## Methods

### Study design and sampling

This qualitative study is part of a hybrid type II implementation-effectiveness trial (Wellbeing While Waiting) evaluating social prescribing in CAMHS. The full trial procedures have been published in a study protocol [18]. This paper reports on qualitative interviews conducted with the control group, with intervention findings to be reported separately. Ethics approval for the trial was obtained from NHS Research Ethics Committee (Ref 22/WS/0184).

Staff at ten participating CAMHS sites across England recruited eligible participants to the control group of the main trial. Youth were eligible for the trial if they were aged 11–18 and had been on the CAMHS waitlist for less than one month for a short-term evidence-based treatment. Youth were excluded if they had eating disorders, psychosis, or severe and complex difficulties (as determined by CAMHS clinicians) as these typically require more intensive treatment and have shorter wait times (due to the nature of their difficulties). Within the trial, youth and caregivers (of youth under 16 years old) were asked if they would agree to take part in an optional interview after six-month follow-up. Study invitations explained that the interviews aimed to understand the experiences of youth on waiting lists before the roll-out of social prescribing at CAMHS sites. Participants were made aware that participation (or non-participation) would not affect their CAMHS treatment, and the findings could help to inform future improvements to mental health services. Each interviewee was also offered a £10 voucher in appreciation of their time and contributions. Youth aged 16–18 provided informed consent to participate in interviews. Youth aged 11–15 gave assent and caregivers gave informed consent for their child’s participation as well as their own participation in interviews.

In total, 216 youth were recruited to the control group. Of 117 youth and 92 caregivers who consented to being contacted for interviews, 85 youth and 63 caregivers were invited to take part via email or phone call (see Supplementary Appendix 1 for more information). Purposive sampling was used to capture maximum variation across sites, demographic characteristics, and self-reported mental health difficulties, ensuring that the sample reflected the diversity of youth on CAMHS waitlists. 11 youth and five caregivers declined to take part because they were not interested or comfortable with being interviewed. Another 54 youth and 43 caregivers did not respond to invitations, providing a final sample of 20 youth and 15 caregivers. Although youth aged 11–18 were eligible to take part, those who participated in interviews were aged 11–17.

### Data collection and analysis

Semi-structured interviews were conducted by EH, ABr, ABu, or JW using a topic guide designed to explore waiting experiences, coping strategies, and views on social prescribing (Supplementary Appendix 2). The data on social prescribing are not reported in this paper and are being analysed separately. All interviewers had experience conducting interviews on sensitive topics with patient and caregivers, including youth with mental health difficulties. All interviews followed the questions in the guide for consistency, while language was tailored for participant age (e.g., using phrases like “make yourself happy or well” instead of “improve your mental health”) and using terms that each interviewee was familiar with (e.g., “CAMHS”, “mental health services”, “support”, or “treatment”). Interviewers also followed a standard protocol for minimising and managing distress, including reminding participants that they could skip questions, take a break, or withdraw at any time without any reasons or consequences, and offering follow-up support if needed (including signposting to national and site-specific support sources).

Youth and caregiver interviews took place separately – youth could take part in an interview even if their caregivers did not take part an vice versa, so not all interviewees formed parent-child dyads. Interviews took place over Microsoft Teams or telephone (depending on participants’ preferences) and lasted between 20 and 60 min (averaging 30 min). All interviews were audio- or video-recorded, de-identified, and transcribed verbatim by the respective interviewers. Information was also collected on youth’s and caregivers’ age, gender, and ethnicity, as well as youth’s self-reported mental health difficulties. Data collection ceased when interviewers agreed that the sample was diverse and large enough to provide adequate “information power” [19] to answer the research questions, but not too large to dilute in-depth analysis of individual participant accounts.

As interview data was rich and nuanced, reflexive thematic analysis was undertaken. The analytic process followed six steps: data familiarisation, coding, generating initial themes, reviewing themes, refining themes, and writing up [20]. EH coded all the transcripts in NVivo and developed initial themes inductively, focusing on patterns of shared meaning relevant to the research aims while ensuring that differing experiences were considered. Youth and caregiver transcripts were analysed together to generate convergent, complementary, or divergent themes between the two stakeholder groups. Convergent themes referred to areas where youth and caregivers shared similar views; complementary themes captured insights raised by only one stakeholder group that added depth without contradicting the other group’s account; and divergent themes referred to direct contrasts in perspectives between groups. The other authors familiarised themselves with a subset of transcripts that were selected to reflect a range of sites, participant characteristics, and experiences, and participated in two discussions to reflect on their understanding of the data and its consistency with the themes generated by EH. These discussions ensured that no significant areas of meaning had been overlooked and informed refinement of theme names and boundaries, with decisions ultimately grounded in EH’s analysis of the full dataset. All authors reached an agreement on the final themes.

### Patient and public involvement

A Youth Advisory Group (YAG) comprising six young people (aged 14–22) with lived experience of mental health difficulties gave input into the study design and documents. This included ensuring that questions in both the demographic surveys and qualitative interviews were age-appropriate, accessible, and resonated with youth experiences. For example, the categories of self-reported mental health difficulties used in the survey were based on the DSM [21] but co-developed with the YAG to make them more youth-friendly (e.g., ‘ADHD’ was rephrased as ‘behavioural difficulties – difficulties sitting still and concentrating’). In addition, two youth lived experience researchers contributed to qualitative analysis by reviewing codes and providing feedback on the interpretation of themes.

## Results

Between April to September 2024, 20 youth and 15 caregivers took part in interviews, comprising 10 caregiver-youth dyads, as well as 10 youth and 5 caregivers who participated independently (Table 1). Characteristics of the full control group sample can be found in Supplementary Appendix 3.

**Table 1:** Participant characteristics

Characteristic	Youth (*n* = 20)		Caregivers (*n* = 15)	
Geographical region				
South West England	2		0	
London and South East England	2		2	
North East England	5		5	
North West England	2		1	
East of England	4		4	
West Midlands	5		3	
Age				
	11 years	3	< 35 years	0
	12 years	2	35–39 years	5
	13 years	2	40–44 years	4
	14 years	5	45–49 years	4
	15 years	3	50–54 years	1
	16 years	2	55–59 years	1
	17 years	3	> 59 years	0
Gender				
Male	7		2	
Female	12		13	
Non-binary	1		0	
Ethnicity				
White	17		14	
Black, African, Caribbean or Black British	1		1	
Asian or Asian British	0		0	
Mixed or multiple ethnic groups	2		0	
Other ethnic groups	0		0	
Self-reported mental health difficulties*				
Developmental difficulties	2		N/A	
Eating difficulties	5		N/A	
Mood difficulties	18		N/A	
Behavioural difficulties	11		N/A	
Personality difficulties	7		N/A	
Seeing or feeling things which are not there	4		N/A	
Anxiety difficulties	17		N/A	
Substance use	1		N/A	
Self-harm	8		N/A	

*Some youth reported multiple mental health difficulties. As youth with eating disorders or psychosis were excluded from the study, difficulties with eating and seeing or feeling things that are not there could reflect symptoms of other presenting problems (e.g., loss of appetite due to anxiety, or trauma-related experiences). While some caregivers also spoke about their own mental health during interviews, they were not asked to report specific mental health difficulties

**Figure 1:**
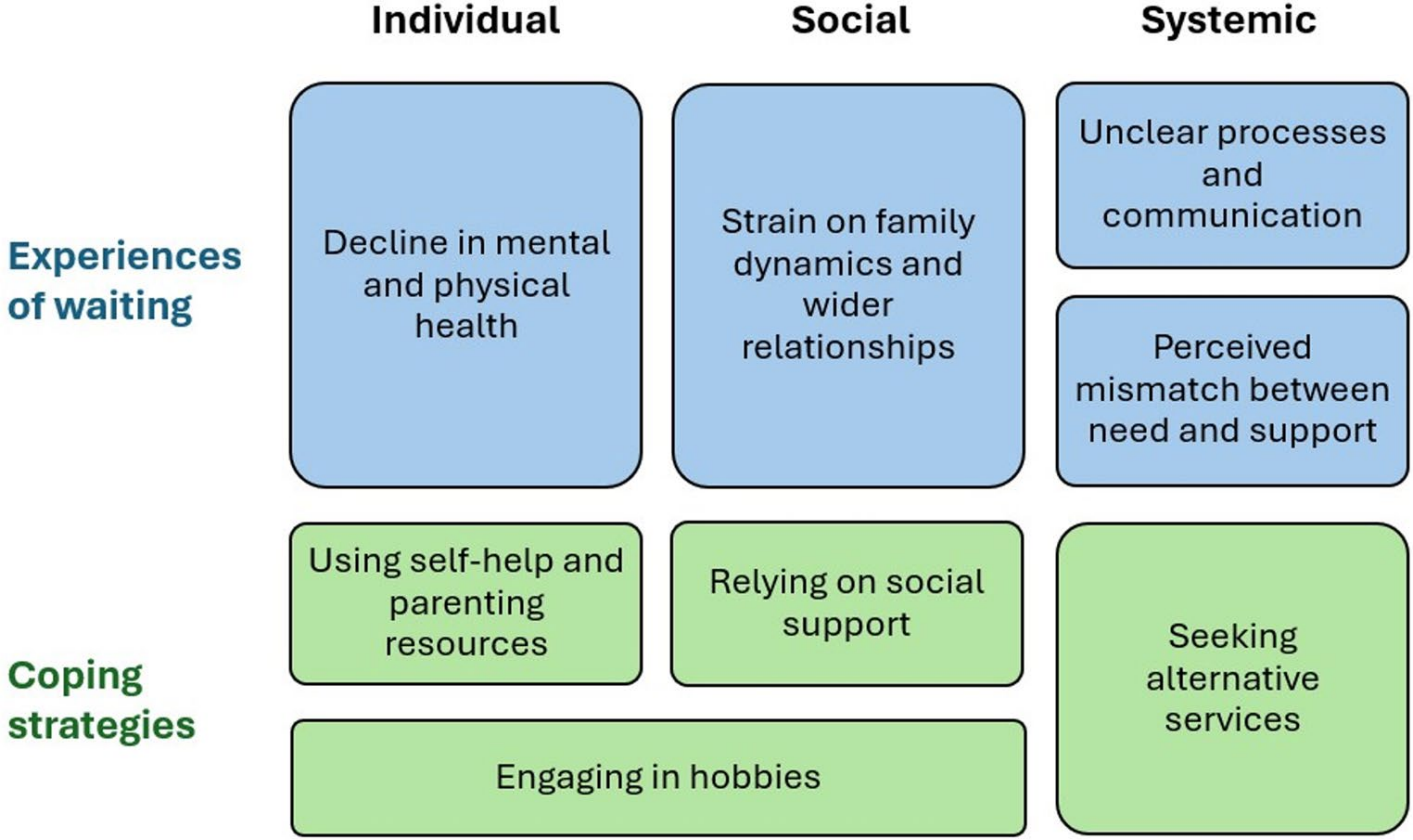
Fig 1. Main themes

Eight main themes were generated from the interview data, illustrating experiences of waiting and coping strategies across the *individual*,* social*,* and systemic levels* (Fig. 1). Convergent themes across youth and caregiver data included a decline in mental and physical health, unclear processes and communication, and perceived mismatch between need and support. However, some younger participants had less insight into referral and waiting processes, which were often managed by their caregivers, while older youth and caregivers provided more detailed accounts of service navigation and communication with CAMHS. Strain on family dynamics and wider relationships was a complementary theme raised only by caregivers and not explicitly mentioned by youth themselves. Common coping strategies identified by both groups included using self-help and parenting resources, relying on social support, engaging in hobbies, and seeking alternative services, although opinions sometimes diverged around the perceived value of these strategies (e.g., youth tended to view hobbies and school-based support positively, whereas caregivers occasionally expressed concern that these activities could interfere with their child’s education). Supporting quotes from participants are provided throughout the text and in Supplementary Appendix 4. ID numbers of youth and caregivers are unlinked to preserve pseudonymity.

### Decline in mental and physical health

Some participants reported that delays in accessing support prolonged youth’s existing mental health symptoms without improvement, while others expressed that waiting not only prolonged but exacerbated symptoms including anxiety, depression, and suicidal thoughts and behaviours:

“I got angry at myself more often. And loads of intrusive thoughts, self-harm and I think it did get worse.” (Youth_009).

Most concerningly, for some youth, being put on a waitlist actively triggered a decline in mental and physical health:

“From that appointment, [my child]’s mental health just took a complete nosedive, so she stopped eating. And I think it was directly correlated to that because she took the view that I’ve just told them everything that was upsetting me, but they don’t want to help me, and they can’t help me. So, what’s the point? I can’t wait that long. So, she kind of didn’t want to live anymore.” (caregivers_023).

As this youth became severely underweight, “the eating disorder team got involved” and shortened the wait, but her parent alongside other caregivers interviewed felt that such deterioration could have been avoided if they had received support earlier. Caregivers felt “fearful” and “worried” about their child’s worsening mental state, and “guilty” and “useless” for not being able to help their child, which affected their own mental health and pre-existing physical conditions:

“Any stress I get affects my ME^1^ and what’s happening now is it’s also affecting my diabetes. So, my diabetes is going up and… that then has implications on my health. But the doctors have agreed that it’s not from what I’m eating, it’s from stress. It’s all from stress.” (Caregiver_034)

### Strain on family dynamics and wider relationships

Caregivers described a shift in family dynamics while waiting, as the onus was on them to “fill the void” left by the lack of professional mental health support, alongside having to work and care for other children. The increased attention paid to the youth on the waitlist often caused tension within families:

“[Sibling’s name] was struggling with us trying to help [youth’s name]. Obviously, she’s getting a lot of attention, so it did cause a lot of problems within the family… It caused a lot of arguments within the family between the girls.” (Caregiver_024).

Untreated mental illness also led to youth becoming “more isolated”, “withdrawn”, and “struggling to attend school”, which had “knock-on effects” on their siblings’ school lives and social lives:

“Because things did escalate, a few weeks ago, even [sibling’s name] was really struggling in school. She ended up coming home from school because she just found it so difficult… friends look at her and think well, why are you going off… and it has unfortunately affected her friendships as well.” (Caregiver_032).

### Unclear processes and communication

Both youth and caregivers found the process of accessing mental health services “confusing” and “disjointed”. They recalled receiving “numerous calls from different mental health services” without clear communication on which was provided by CAMHS:

“We just didn’t understand why a school nurse was ringing us. We didn’t understand why we were getting an emotional support worker… I did come away sort of thinking I’m not really sure who you’re affiliated with and where you’re coming from.” (Caregiver_021).

Participants also described convoluted referral pathways and re-referrals to CAMHS, which entailed a series of waits even before they were put on the waitlist:

“I’ve been through it twice because of my high school not doing the right things… The first time it wasn’t done correctly… I called up again and was like, right. I really need help. And then I was put on the waiting list.” (Youth_019).

After being put on the waitlist, most participants reported receiving inadequate or inconsistent information about wait times, making them feel “uncertain”, “unsettled”, and unable to plan their lives. Caregivers expressed the frustration of having to “chase”, “fight”, and “hound” for information, and still not being able to obtain a clear answer:

“If you know, OK, right, I’ve got three months, I suppose you can put that in place. You’ve got some hope haven’t you. Yeah, but if you just don’t know anything, it could be a year. You just constantly feel like you don’t know what’s happening, or if you’re even gonna get a service.” (Caregiver_026).

Only two participants reported receiving regular updates on their waitlist status, which helped to manage expectations:

“We got a few messages trying to keep us updated. We got a message say at 3 months saying it would be 5 months, then at 5 months, I think it’d be 6… which I found was quite helpful to know what’s actually going on.” (Youth_006).

### Perceived mismatch between need and support

Youth shared that it took time for them to “accept that they’re struggling with their mental health”, “build up the courage” to seek help, and undergo the lengthy referral process, so by the time they were assessed by CAMHS, they “needed help there and then”. Yet, youth described being left on the waitlist for months or years with nothing except a crisis line telephone number. Caregivers also recounted receiving dismissive or unhelpful responses even during crises, such as being told “just carry on doing what you’re doing”, “you still have to wait”, or “go to A&E”:

“I rang up, because he cut his arm. I never heard back from them. They said, oh, when the caseworker is free, we’ll ring you back. Well, they never rang back. So, there’s just like no faith in that service… In the six years since he’s been on [the waitlist], he’s only ever had one catch up phone call. Maybe if there was more of that, just have someone to check is everything OK? Is there anything we can do, or has anything changed?” (Caregiver_031).

Lack of support and contact from CAMHS while waiting made participants feel “alone”, “forgotten” and “let down” by services, making it difficult for some youth to trust professionals when they were eventually seen:

“I think, her not hearing anything and constantly the weeks going on and not having any link or contact at all was more damaging for her really, because it’s almost like, ‘these people don’t care’. And now when she has actually met her CPN^2^, basically, for every time she’s seen him, he’s getting the brunt of, ‘you ignored me. You’re not doing anything.’” (Caregiver_032)

In addition to the lack of interim support, some youth were put on waitlists for unwanted or unsuitable services. Participants reported that professionals disregarded their opinions regarding the cause of their mental health difficulties and preferred treatment:

“I was telling them I don’t want family therapy. I don’t think it’s got anything to do with my family and stuff. But they were like, well, that’s what we want.” (Youth_011).

Waiting a long time for support that was perceived to be inappropriate made youth and caregivers feel that their wait was “pointless” or a “waste of time”, leaving them “even more desperate” and “deflated”. Some participants ultimately declined support when it became available and “gave up” or were put back on the waitlist for other types of support.

### Using self-help and parenting resources

While waiting for formal services, participants used mental health websites, apps, and books to learn self-help or parenting tools. Sometimes, CAMHS provided such resources, but caregivers reported that a level of independent research was still needed. This could lead to conflicting information and make it “difficult to know what to do for the best”:

“I think [CAMHS] just directed us to a website and to say, we’ve got a lot of information, and you can search that up yourself… we did a little bit of research and just self-googling, which isn’t always, you know helpful.” (Caregiver_033).

While methods such as “mindfulness”, “breathing techniques”, and “relaxation exercises” helped to provide temporary relief, participants stressed that they were not long-term solutions or substitutes for professional support:

“I mean, she did have methods that she picked up from [name of mental health charity], which was really, really good. But obviously that had only worked to a certain extent, otherwise she wouldn’t have needed CAMHS… Yeah, it wasn’t enough to settle her permanently. It was just something to help her get by now and again.” (Caregiver_035).

Some caregivers also struggled to get their youth to engage with these resources and felt “an external person” was needed to help them, explaining that they had turned to CAMHS precisely because their own strategies were “not working anymore”.

### Engaging in hobbies

Participants engaged in a variety of hobbies such as physical activities (e.g., sports, exercise) and creative activities (e.g., music, arts and craft) while on the waitlist. Youth and caregivers said that these recreational activities supported their wellbeing by providing a form of distraction, a sense of achievement, and/or stress relief:

“Drawing… I think it’s very relaxing and when you start it… it’s like a goal to finish it. And then when you do finish it, you feel good.” (Youth_002).

“She likes singing and especially with piano, every time that she’s very stressed and anxious, she gives up what she’s doing and plays a little bit on the piano and she feels relief for a while before she carries on. Yeah, that’s what helps her a lot.” (Caregiver_029).

However, one caregiver was concerned that their child’s strong attachment to their hobby led to neglect of schoolwork, and one youth felt that hobbies were not enough to address their specific mental health needs:

“They kind of just tell you to find a hobby or something. And then they just ended it… but for some children, it’s not about hobbies… It’s about their mental state and trauma or what they need to be helped with.” (Youth_016).

### Relying on social support

While waiting to see a mental health professional, some youth talked to their friends, family, and other trusted adults, which they found helpful to varying degrees:

“I’d speak to some of my close friends. One girl in particular, she’s amazing and she’s known me all her life… And my other friend who saved me from probably my darkest point, I’d say.” (Youth_018).

“I’d try to talk to my parents and my friends, but not many of them would kind of understand.” (Youth_014).

Lack of understanding from their existing social networks led some Youth and caregivers to seek support groups where they could meet others with shared experiences. For example, one caregiver found it helpful to attend a caregivers’ meeting at her local mental health hub, although this was not an ongoing source of support due to clashing work commitments:

“I did attend one and I must admit that was helpful to feel that, yeah, actually, I’m not on my own here. There are other people going through this. Feeling the same way.” (Caregiver_024).

### Seeking alternative services

Given the limitations of informal support, some participants sought help from services outside of CAMHS, including school, charity, and private services. Youth particularly valued having a school counsellor they could talk to “in the moment”:

“I was seeing a counsellor every week at school for a few weeks, and then that carried on until half term… yeah, I found that helpful. If anything went wrong, then I knew I had someone to talk to.” (Youth_017).

However, one caregiver felt that having counselling at school affected their child’s studies, and other participants reported that alternative services were also plagued with problems like lack of continuity:

“The appointments were only half an hour. And I’ve had to speak to someone new every time. So, it’s like every time I speak to them, I’d have to explain everything that was going on. And by the time I’d explained everything, there was 5 minutes left. So, I couldn’t really get any help at all.” (Youth_011).

## Discussion

This study provides the first in-depth exploration of how CAMHS waitlists impact youth aged 11–17 and their parents and families. Our findings largely align with quantitative studies showing that most youth experience a deterioration in mental health while waiting for support, although a minority may report symptom improvement [8–11]. However, in our study, no participants mentioned symptoms subsiding during the wait. Instead, all described their symptoms either worsening or persisting, with some perceiving their exacerbated symptoms to be “a direct result of waiting” (as described by a youth). Furthermore, and concerningly, untreated mental illness and stress associated with waiting have knock-on effects on the physical health and wellbeing of the whole family, carrying implications not only for CAMHS but also other parts of the healthcare, third sector, and educational systems.

Multiple aspects of the design of waitlists are responsible for these problems. One aspect is unclear information throughout the referral and waiting process, as well as inappropriate services following long waits, which corroborates a qualitative study conducted with older adolescents during the pandemic [22], but shows the problem is ongoing. Another aspect is that youth and caregivers experience a stark lack of contact and support from CAMHS while waiting despite their perceived need for urgent help, leaving them feeling alone, neglected, and disillusioned with services. These findings could be shaped by the medicalisation and professionalisation of mental health support in the UK, where responsibility for mental health is placed primarily within the domain of clinical expertise [23]. Like previous research conducted in the UK [22], we found that CAMHS was often perceived by youth and caregivers as the pinnacle of mental health support, creating high expectations and intensifying distress when specialist care was not received as hoped. While participants made active efforts to manage their symptoms by turning to alternative services, social support, hobbies, and self-help resources, limitations to these coping strategies suggest that much more needs to be done to support youth with mental health difficulties and their families.

Based on our findings, we make four key recommendations for policy and practice:

1. Shorten CAMHS waiting times.

Our findings highlight that CAMHS should be prioritised in the new government’s pledge to shorten NHS waitlists [24], as waiting is not only harming youth but also having detrimental effects on families. Underinvestment in community-based services [2] and severe shortages in psychiatrists [25] have been identified as key drivers of long waits for mental health services in the UK, pointing to the need for both upstream and downstream strategies to reduce CAMHS waitlists.

We echo recommendations by ESCAP and the Darzi report to increase investment in mental health promotion, prevention, and early intervention programmes [2, 17]. This should include investing in developing the evidence base for promising interventions such as social prescribing [26] so these can be scaled up widely if proven to be effective. Lessons can also be drawn from European countries such as Finland and Norway, which report among the lowest levels of unmet youth mental health needs [27]. Both systems have successfully implemented decentralised mental health support embedded in community and school settings, with welfare services provided to all students by specially trained professionals (e.g., school nurses, counsellors) and a strong curricular focus on mental health literacy and wellbeing [27, 28]. Although the UK has started to introduce mental health support teams in schools, roll-out has been unevenly distributed across the country and is projected to reach only 60% of students by 2026, with nationwide coverage planned by 2030 [29]. Given that many youth in our study valued school-based support while waiting, piloting upstream programmes that promote positive development, resilience and psychosocial wellbeing, such the Finnish “Let’s Talk About Children” intervention [30], may help to reduce downstream reliance on CAMHS.

Concurrently, preventative efforts should be supplemented by enhancing the capacity and efficiency of CAMHS to provide specialised treatment for youth with more complex needs. CAMHS sites that introduced a single point of access [31] or the Choice and Partnership Approach (CAPA) [32] have reported that such models can streamline referral processes and reduce waiting time to first contact, but can also lead to internal waits and bottlenecks without sufficient staffing resources to manage demand. Thus, expanding and sustaining the mental health workforce is essential to improve patient flow through CAMHS alongside initiatives to facilitate quick access [33]. Indeed, the success of Finland and Norway’s community-based models is supported by their relatively high ratios of child and adolescent psychiatrists per child [27]. Additionally, the potential of virtual and hybrid models of care warrants further exploration, especially in underserved regions. Evidence from the pandemic suggests that youth and caregivers have had mixed experiences with remote CAMHS appointments, appreciating their flexibility and accessibility while highlighting significant drawbacks compared to in-person delivery, underscoring the need for careful consideration around implementation [22, 34].

2) Improve information sharing and communication.

While shortening waitlists should be the ultimate goal, experiences of waiting could also be improved with clearer communication and information sharing. Our findings show that people appreciate transparency and generally show understanding when information is shared up front, even when wait times are being revised. Research on physical health services have suggested that proactively informing patients about delays can reduce uncertainty, enhance a sense of control, and make patients perceive the wait as more manageable or tolerable [35, 36]. This recommendation is even more pertinent to mental health services, given that uncertainties related to the wait can intensify existing symptoms of anxiety and stress as well as lead to new symptoms emerging [16]. Once youth are referred to CAMHS, information should be provided on next steps and estimated wait times, and subsequent follow-ups should be made to update patients on waitlist status. Information sharing between service providers also needs to be improved, such as when GPs or schools refer youth to CAMHS, to avoid lengthening referral processes.

3) Provide interim support.

Interim support is vital for mitigating against mental health deterioration and feelings of isolation or neglect while waiting for formal services. As suggested by our participants, this can take the form of periodic phone calls to ‘check in’ and respond to any changes in risk presentation. Importantly, our findings emphasise the value having a single point of contact (e.g., mental health nurse, case manager) to facilitate continuity of care and building of trust during the waiting period. Furthermore, the use of hobbies as a coping strategy by youth on the waitlist could be directly supported by offering social prescribing referrals that connect youth to community activities based on their interests [26], although our findings also indicate a need to identify which youth this approach would be appropriate for. Additionally, families may benefit from training courses, guided self-help interventions, and peer support groups where they can connect with others on the waitlist and learn coping strategies [37, 38]. It is imperative that support options are offered as early as possible before mental health needs escalate, bearing in mind that assessments and being put on a waitlist can trigger strong emotional and psychological responses [16].

4) Tailor services to patients’ needs and preferences.

Finally, the mismatch perceived by our participants between their mental health needs and the services provided by CAMHS points to the importance of giving youth choice and control over the care they receive. CAPA not only has the potential to reduce wait times as aforementioned, but can also improve patients’ experiences by defining mental health problems from their perspectives and collaboratively deciding on treatment plans [39]. Even if implementing CAPA across all CAMHS sites is not feasible [32], the underlying principles of shared decision-making and person-centred care should be embedded within existing systems. Respecting the views and preferences of youth not only aligns with UK government policy [40] and the United Nations Convention on the rights of the child [41], but can also improve treatment engagement and outcomes [42].

### Strengths and limitations

A strength of this study is the purposeful sampling approach, reflected in the spread of participants across ages and geographical regions of England. Geographical representation was an important sampling domain due to known regional disparities in CAMHS access and wait times, although we did not identify any consistent differences in experiences between sites. The range of mental health difficulties reported by our participants also capture primary referral reasons to CAMHS in 2022–2023 [43]. Additionally, this study included the voices of younger adolescents, who have been underrepresented in past research. Although some younger participants had limited awareness of waitlist processes compared to older adolescents, they still shared meaningful reflections on how waiting affected their daily lives and wellbeing. Triangulating youths’ perspectives with caregivers’ perspectives provided complementary information on the wider impact of waiting on the family and some diverging opinions on the helpfulness of coping strategies. A limitation of this study is that our sample had more White and female participants, although this reflects the demographics entering waitlists at CAMHS more generally [43]. Our sample also excludes youth who may have sought and waited for support from CAMHS but were not accepted onto the waitlist. Furthermore, many of the youth and caregivers approached for interviews did not respond or declined to take part. It is possible that these participants had different experiences to those who were interviewed, but it is notable that there were no obvious socio-demographic or diagnostic differences between those who participated and those who did not (Supplementary Appendix 3). The experiences of our interviewees were largely negative, but conscious efforts were made during the analysis and synthesis process to ensure that any positive experiences were also considered and reflected in this write-up, such as experiences of receiving adequate information and developing useful coping strategies.

## Conclusion

This qualitative study has provided novel insights into CAMHS waitlist experiences and coping strategies among youth and caregivers in the UK. The findings highlight the mental and physical health deterioration, relational strain, perceived lack of support, and loss of trust in services that can occur during this period, as well as the resilience and resourcefulness demonstrated by families in supporting their wellbeing and seeking alternative forms of help. Collectively, these insights emphasise the importance of shortening wait times, improving communication, increasing interim support, and individualising care provision to reduce the adverse impact of waiting and ensure that youth receive timely and appropriate help for their mental health.

## Supplementary Information

Below is the link to the electronic supplementary material.

Supplementary Material 1 (DOCX 29.1 KB)

Supplementary Material 2 (DOCX 26.1 KB)

Supplementary Material 3 (DOCX 20.1 KB)

Supplementary Material 4 (DOCX 20.1 KB)

## Data Availability

As per ethics approval, raw qualitative data cannot be shared due to containing information that might compromise the identity of research participants.

## Footnotes

1 Myalgic encephalomyelitis, also known as chronic fatigue syndrome.

2 Community Psychiatric Nurse.
