# Supplementary material for: Experiences of youth and caregivers waiting for mental health services in the UK: a qualitative study to inform policy and practice
Source: Eur Child Adolesc Psychiatry. 2026 Jan 5;35(5):1467–77. doi: 10.1007/s00787-025-02952-x (PMC13272595; doi:10.1007/s00787-025-02952-x)
Supplement: Supplementary file 1 — Supplementary Material 1 (DOCX 29.1 KB) [file 787_2025_2952_MOESM1_ESM.docx]

**Appendix 1. Consolidated criteria for reporting qualitative studies (COREQ): 32-item checklist**

| **No. Item** | **Guide questions/description** | **Reported on Page #** |
| --- | --- | --- |
| **Domain 1: Research team and reﬂexivity** |  |  |
| *Personal Characteristics* |  |  |
| 1. Inter viewer/facilitator | Which author/s conducted the interview or focus group? | EH, ABr, ABu, JW |
| 2. Credentials | What were the researcher’s credentials? E.g. PhD, MD | EH: PhD, MSc, BA  ABr: MA, BA  ABu: PhD, MSc,BSc  JW: MSc, BSc |
| 3. Occupation | What was their occupation at the time of the study? | EH: Post-doctoral research fellow  ABr: Research Programmes Manager / Research Fellow  ABu: Senior Research Fellow  JW: Research Assistant |
| 4. Gender | Was the researcher male or female? | EH: Female  ABr: Female  ABu:Female  JW: Female |
| 5. Experience and training | What experience or training did the researcher have? | EH: experience conducting qualitative interviews with a range of patient and caregiver groups  ABr: experience conducting qualitative interviews with a range of patients and health professionals ABu: experience of conducting interviews with young people, parents and adults experiencing mental health difficulties  JW: experience conducting qualitative interviews with young people with mental health difficulties |
| *Relationship with participants* |  |  |
| 6. Relationship established | Was a relationship established prior to study commencement? | No |
| 7. Participant knowledge of the interviewer | What did the participants know about the researcher? e.g. personal goals, reasons for doing the research | Interviewers explained to participants that the interviews aimed to understand their experiences of waiting to inform future improvement of mental health services and support for young people and their families. |
| 8. Interviewer characteristics | What characteristics were reported about the inter viewer/facilitator? e.g. Bias, assumptions, reasons and interests in the research topic | EH: none reported  ABr: none reported  ABu: none reported JW: none reported |

| **Domain 2: study design** |  |  |
| --- | --- | --- |
| *Theoretical framework* |  |  |
| 9. Methodological orientation and Theory | What methodological orientation was stated to underpin the study? e.g. grounded theory, discourse analysis, ethnography, phenomenology, content analysis | Reflexive thematic analysis (page 4) |
| *Participant selection* |  |  |
| 10. Sampling | How were participants selected? e.g. purposive, convenience, consecutive, snowball | Purposive sampling (page 4) |
| 11. Method of approach | How were participants approached? e.g. face-to-face, telephone, mail, email | Participants were first approached via email or telephone depending on their contact preferences. A follow-up call was also made to those who did not respond to two emails. A maximum of three contact attempts were made for each participant. |
| 12. Sample size | How many participants were in the study? | 35 (20 CYP and 15 parents) |
| 13. Non-participation | How many people refused to participate or dropped out? Reasons? | 11 CYP and 5 parents declined to take part because they were not interested in or comfortable with being interviewed. 54 CYP and 43 parents did not respond to interview invitations. |
| *Setting* |  |  |
| 14. Setting of data collection | Where was the data collected? e.g. home, clinic, workplace | Remotely via Microsoft Teams or telephone |
| 15. Presence of non-participants | Was anyone else present besides the participants and researchers? | No |
| 16. Description of sample | What are the important characteristics of the sample? e.g. demographic data, date | Reported on pages 5-6 of main manuscript |
| *Data collection* |  |  |
| 17. Interview guide | Were questions, prompts, guides provided by the authors? Was it pilot tested? | Interviews were semi-structured based on a topic guide provided in the supplementary materials |
| 18. Repeat interviews | Were repeat interviews carried out? If yes, how many? | No |
| 19. Audio/visual recording | Did the research use audio or visual recording to collect the data? | Interviews were audio- or video-recorded depending on participants’ preferences. |
| 20. Field notes | Were ﬁeld notes made during and/or after the interview or focus group? | No |
| 21. Duration | What was the duration of the inter views or focus group? | 20-60 minutes |
| 22. Data saturation | Was data saturation discussed? | The concept of data saturation is not considered appropriate within reflexive thematic analysis. Instead, the concept of information power was discussed (page 4). |
| 23. Transcripts returned | Were transcripts returned to participants for comment and/or correction? | No |
| **Domain 3: analysis and ﬁndings** |  |  |
| *Data analysis* |  |  |
| 24. Number of data coders | How many data coders coded the data? | EH coded the data with input from the rest of the research team (pages 4-5). |
| 25. Description of the coding tree | Did authors provide a description of the coding tree? | Yes (page 6) |
| 26. Derivation of themes | Were themes identiﬁed in advance or derived from the data? | Themes were developed inductively from the data (pages 4-5). |
| 27. Software | What software, if applicable, was used to manage the data? | NVivo |
| 28. Participant checking | Did participants provide feedback on the ﬁndings? | No |
| *Reporting* |  |  |
| 29. Quotations presented | Were participant quotations presented to illustrate the themes/ﬁndings? Was each quotation identiﬁed? e.g. participant number | Quotes with participants numbers are presented in the Panel and throughout the narrative text (pages 6-13). |
| 30. Data and ﬁndings consistent | Was there consistency between the data presented and the ﬁndings? | Yes (pages 6-13) |
| 31. Clarity of major themes | Were major themes clearly presented in the ﬁndings? | Yes (pages 6-13) |
| 32. Clarity of minor themes | Is there a description of diverse cases or discussion of minor themes? | Yes (pages 6-13) |
