# Supplementary material for: Experiences of youth and caregivers waiting for mental health services in the UK: a qualitative study to inform policy and practice
Source: Eur Child Adolesc Psychiatry. 2026 Jan 5;35(5):1467–77. doi: 10.1007/s00787-025-02952-x (PMC13272595; doi:10.1007/s00787-025-02952-x)
Supplement: Supplementary file 2 — Supplementary Material 2 (DOCX 26.1 KB) [file 787_2025_2952_MOESM2_ESM.docx]

**Appendix 2. Interview topic guides**

**Interview topic guide for children and young people in the control group**

**I. Opening**

I would like to ask you some questions to learn more about your experiences of being on a waiting list to be seen by a mental health professional. We hope to use this information to understand whether there are ways to support young people in the future. The interview should last less than an hour, and with your permission, I would like to audio record the interview. Would this be okay? Do you have any questions before we begin?

**II. The initial referral**

1. Can you tell me about your recent experiences of being referred to mental health services *(PROMPT: Who referred you (parent/GP/teacher), were you given any information, did you see anyone at the beginning like a psychologist or therapist or support worker or someone from CAMHS?)*

**III) Experiences of waiting**

1. Can you tell me about your experiences while waiting to be seen by a mental health professional? (*PROMPT: how long did you have to wait? Was it explained to you that you would have to wait and for how long?*

1. How did it feel to be on a waiting list?

1. Did being on a waiting list impact you in any way? If yes, in what way? *(PROMPT: mental health, social life and relationships, school activities, physical health)*

**IV) Coping strategies to support mental health**

1. While you were waiting to be seen by a mental health professional, what kind of things did you do to make yourself happy or well? *(PROMPT: activities, services, treatments, people you would talk to)*
2. Did you find any of those things helpful? **If yes** why did they help? **If not**, why not?
3. Were you offered any other support while you were waiting to be seen by a mental health professional? If yes, what types of support were offered? *(PROMPT IF UNCLEAR: Was this support offered by the health service, if not, who offered this support?)*

**V. Views on Social Prescribing**

a) Can you tell me what you understand, if anything at all, about social prescribing? *(NOTE: If the CYP does not know what social prescribing is – researcher to summarise it to them)*

b) Would you be interested in taking part in social prescribing? *(PROMPTS: Why/why not, what might you need to know about social prescribing to help you decide/to make it more appealing?* *What activities, if any, might you be interested in doing?)*

c) Do you feel that being able to access social prescribing while you were waiting for mental health services would have been helpful? *(PROMPTS: Can you tell me why/ why not? What activities, if any, might have been beneficial for your mental health? How might this have helped you?)*

d) What, if any, barriers, or difficulties might you have in taking part in social prescribing? *(PROMPTS: any difficulties in attending appointments/activities, timing of appointments and activities, motivation to engage, knowledge about available activities, views of family or friends)?*

e) What might help you, if anything at all, to take part in social prescribing?

**VI Future changes**

1. Is there anything else that would have helped you while you were waiting to be seen by a mental health professional? If yes, what would have been helpful?

**VII. Closing**

I appreciate the time you took for this interview. Is there anything else that we have not covered that you would like to add about your experiences of being on a mental health waiting list?

**Interview topic guide for parents/guardians of children and young people in the control group**

**I. Opening**

I would like to ask you some questions to learn more about your experiences of being on a waiting list to be seen by a mental health professional. We hope to use this information to understand whether there are ways to support young people in the future. The interview should last less than an hour, and with your permission, I would like to audio record the interview. Would this be okay? Do you have any questions before we begin?

**II. Demographic information**

1. **Can you tell me your:**

Age

Gender

Ethnicity

Relationship to the child who is participating in the study? (e.g. parent, grandparent, guardian etc)

**III. The initial referral**

1. Can you tell me about your experiences of your child being referred to mental health services? *(PROMPT: Who referred your child, were you/your child given any information, did you/your child see anyone at the beginning?)*

**IV. Experiences of waiting**

1. Can you tell me about your experiences while your child was waiting to be seen by a mental health professional? (*PROMPT: how long did you/your child have to wait? Was it explained to you/your child that they would have to wait and for how long?)*

1. How did it feel to have your child on a waiting list?

1. Did your child being on a waiting list impact you in any way? If yes, in what way? *(PROMPT: mental health, physical health, social life and relationships)*
2. Do you think being on a waiting list impacted your child in any way? If yes, in what way? *(PROMPT: mental health, physical health, social life and relationships, school activities)*

**V. Coping strategies to support mental health**

1. While your child was waiting to be seen by a mental health professional, what kind of things did you do to support your mental health? *(PROMPT: activities, services, treatments, people you would talk to)*
2. Did any of those things help you? **If yes,** why did they help? **If not**, why not?
3. While your child was waiting to be seen by a mental health professional, what kind of things did they do to support their mental health? *(PROMPT: activities, services, treatments, people they would talk to)*
4. Did you find any of those things helped your child? **If yes,** why did they help? **If not**, why not?
5. Was your child offered any other support while they were waiting to be seen by a mental health professional? If yes, what types of support were offered? *(PROMPT IF UNCLEAR: Was this support offered by the health service, if not, who offered this support?)*

**VI. Views on Social Prescribing**

1. Can you tell me what you understand, if anything at all, about social prescribing? *(NOTE: If the participant does not know what social prescribing is – researcher to summarise it to them)*

b) Would you be interested in letting your child take part in social prescribing? *(PROMPTS: Why/why not, what might you need to know about social prescribing to help you decide/to make it more appealing?* *What activities, if any, do you think your child might be interested in accessing?)*

c) Do you feel that being able to access social prescribing while your child was waiting for mental health services would have been helpful? *(PROMPTS: Can you tell me why/ why not? What activities, if any, might have been beneficial for your child’s mental health? How might this have helped you/your child?)*

d) What, if any, barriers, or difficulties might your child have in taking part in social prescribing? *(PROMPTS: any difficulties in attending appointments/activities, timing of appointments and activities, motivation to engage, knowledge about available activities, views of family or friends)?*

e) What might help your child, if anything at all, to take part in social prescribing?

**VII Future changes**

1. Is there anything else that would have helped you/your child while they were waiting to be seen by a mental health professional? If yes, what would have been helpful?

**VIII. Closing**

I appreciate the time you took for this interview. Is there anything else that we have not covered that you would like to add about your child’s experiences of being on a mental health waiting list?
