# Supplementary material for: Experiences of youth and caregivers waiting for mental health services in the UK: a qualitative study to inform policy and practice
Source: Eur Child Adolesc Psychiatry. 2026 Jan 5;35(5):1467–77. doi: 10.1007/s00787-025-02952-x (PMC13272595; doi:10.1007/s00787-025-02952-x)
Supplement: Supplementary file 3 — Supplementary Material 3 (DOCX 20.1 KB) [file 787_2025_2952_MOESM3_ESM.docx]

**Appendix 3. Comparison of CYP characteristics in qualitative sample and full control group sample**

| **Characteristic** | **CYP in qualitative sample (n=20)** | **CYP in full control group sample (n=216)** |
| --- | --- | --- |
| **Geographical region** | | |
| South West England | 2 (10.0%) | 20 (9.3%) |
| London and South East England | 2 (10.0%) | 19 (8.8%) |
| North East England | 5 (25.0%) | 69 (31.9%) |
| North West England | 2 (10.0%) | 18 (8.3%) |
| East of England | 4 (20.0%) | 41 (19.0%) |
| West Midlands | 5 (25.0%) | 29 (13.4%) |
| **Age** | | |
| 11 years | 3 (15.0%) | 33 (15.3%) |
| 12 years | 2 (10.0%) | 43 (19.9%) |
| 13 years | 2 (10.0%) | 33 (15.2%) |
| 14 years | 5 (25.0%) | 34 (15.7%) |
| 15 years | 3 (15.0%) | 36 (16.7%) |
| 16 years | 2 (10.0%) | 29 (13.4%) |
| 17 years | 3 (15.0%) | 8 (3.7%) |
| **Gender** | | |
| Male | 7 (35.0%) | 63 (29.2%) |
| Female | 12 (60.0%) | 142 (65.7%) |
| Non-binary | 1 (5.0%) | 7 (3.2%) |
| Prefer not to say | 0 (0.0%) | 3 (1.4%) |
| Other | 0 (0.0%) | 1 (0.5%) |
| **Ethnicity** | | |
| White | 17 (85.0%) | 186 (86.1%) |
| Black, African, Caribbean or Black British | 1 (5.0%) | 6 (2.8%) |
| Asian or Asian British | 0 (0.0%) | 4 (1.9%) |
| Mixed or multiple ethnic groups | 2 (10.0%) | 17 (7.9%) |
| Other ethnic groups | 0 (0.0%) | 2 (0.9%) |
| Not reported | 0 (0.0%) | 1 (0.5%) |
| **Self-reported mental health difficulties*** | | |
| Developmental difficulties | 2 (10.0%) | 30 (13.9%) |
| Eating difficulties | 5 (25.0%) | 42 (19.4%) |
| Mood difficulties | 18 (90.0%) | 168 (77.8%) |
| Behavioural difficulties | 11 (55.0%) | 108 (50.0%) |
| Personality difficulties | 7 (35.0%) | 46 (21.3%) |
| Seeing or feeling things which are not there | 4 (20.0%) | 32 (14.7%) |
| Anxiety difficulties | 17 (85.0%) | 184 (85.2%) |
| Substance use | 1 (5.0%) | 6 (2.8%) |
| Self-harm | 8 (40.0%) | 86 (39.8%) |

*Some CYP reported multiple mental health difficulties
