# Supplementary material for: Experiences of youth and caregivers waiting for mental health services in the UK: a qualitative study to inform policy and practice
Source: Eur Child Adolesc Psychiatry. 2026 Jan 5;35(5):1467–77. doi: 10.1007/s00787-025-02952-x (PMC13272595; doi:10.1007/s00787-025-02952-x)
Supplement: Supplementary file 4 — Supplementary Material 4 (DOCX 20.1 KB) [file 787_2025_2952_MOESM4_ESM.docx]

**Appendix 4. Additional quotes supporting each theme**

| **Category** | **Level** | **Theme** | **Quotes from CYP** | **Quotes from PG** |
| --- | --- | --- | --- | --- |
| Experiences of waiting | Individual | Decline in mental and physical health | “[Waiting] wouldn’t help my self-harm, I would keep doing it and yeah, it wasn’t helping my depression or my motivation to do anything really... I've not been able to walk for periods of time 'cause it's made me really exhausted easily... my body is so beat from my mental health that it’s just kind of broken down.” (CYP_016)  “It didn't feel like I was getting any better, and then once it felt like I got like a bit worse.” (CYP_020) | “I think it left her more anxious… the uncertainty in her, not knowing what the next bit entails when she's already anxious and uncertain about everything else in her head.” (PG_035)  “[CYP’s name] was escalating to the point of self-harm... I think part of it is that ‘nobody cares so therefore I'm just going to up the stakes each time,’ isn't it? And [CYP's name] has said, ‘how far does somebody have to go before you actually get help?’... I ended up getting quite a nasty sort of rash under my arm that totally broke down and ended up having to see a doctor a couple of times, which they did actually agree it could be down to exactly what was going on and a stress reaction almost with the body. So yeah, physically it did have its toll.” (PG_032) |
|  | Social | Strain on family dynamics and wider relationships | N/A – only conveyed by PG | “Her sister wants people to come round and play but I don't want people to come into the house. Depending on how her mood is, we know we don't want them to experience it, so literally there is not a bit of our lives that hasn't been impacted. Like my mum no longer really speaks to me because of [CYP’s name].” (PG_023)  “Even family don't understand, extended family because a lot of people just don't know what to say or what to do to help. Yeah, it's very hard. I can see how it can break families up. You know, tension and extra stress, it does cause a lot of problems within families.” (PG_024) |
|  | Systemic | Unclear processes and communication | “I remember being told that there'd be a waiting list of a few months and that they would be in contact with me… I haven’t heard since last year.” (CYP_001)  “I've been put on the waiting list for CBT, but like, there's no updates. Nobody's calling to check what's happening, and I'm just kind of like in the dark about it.” (CYP_019) | “The GP was useless if I'm honest, when they made a referral. CAMHS got back to us and said the GP hasn't actually told them anything that they need to know about [CYP’s name]… so, we actually had to do a self-referral through their website after contacting them, chasing it up.” (PG_033)  “The second time... we felt a bit more positive about it because the information was shared, because the people that we'd spoken to at CAMHS were a little bit more open with us. So, we can then refer that back to [CYP’s name] and say look, we've got an appointment coming soon.” (PG_028) |
|  |  | Perceived mismatch between need and support | “I'd only seen [the mental health professional] for half an hour, and he said that I might have autism or ADHD just because I was fiddling. And I think it's so wrong how mental health supporting places just instantly go to the conclusion that people with anxiety or trauma automatically have autism or ADHD.” (CYP_016)  “I wasn’t offered any other type of support [while waiting]... Maybe like them checking in on me, seeing if I was OK or if something's changed and if I need something. Maybe if there was someone I felt that I could trust and talk to a little bit more, that might have helped a little bit as well.” (CYP_008) | “What I've seen is from past experiences, services just chucking anything at you because they know that they can't give you what is needed. So, they'll just give you something, and I think for children, that can be incredibly confusing. I mean, I think as an adult it's hard enough to know why somebody's offering you a service that you don't really understand whilst you wait, but I think for a child it would be even more confusing.” (PG_021)  “I think it's all just a case of getting things in place early enough. If you can get in place like, you know, an online chat which is safe, someone that they can talk to, things like that. That's brilliant. But it needs to be done earlier, before they get to the point where they can't. Even if it’s like, events where you could go where other people are all in the same similar situation and you can find out numbers or they can teach about what the diagnosis is, how to manage things at home. You don't get any of that... All we get is, you’re on the waiting list, and this is the emergency number for the out of hours team. Or you know, if it really goes wrong, go to A&E, which just isn't an environment you want to go to when you're in that sort of situation.” (PG_026) |
| Coping strategies | Individual | Using self-help and parenting resources | “My parents got us a couple of books and I tried them, but they didn't really help. I think I just needed to talk to someone.” (CYP_014)  “[My school wellbeing officer] has given me loads of websites to look into and apps to deal with either anxiety, stress, or you know just about your whole well-being to help me, so for example if I'm having a panic attack, it'll tell me what to do and how to calm myself down.” (CYP_013) | “For me, [CYP’s name] was self-harming a lot, and I had to just Google it all. And then obviously, when you Google, you get all sorts of links and it all sometimes says different things, and it's difficult to know what to do for the best.” (PG_031)  “When she was younger, you know, we did try a couple of different things where she kept a diary and wrote down what happened and how she felt and stuff like that. But she's just not interested in it at all now…” (PG_034) |
|  | Individual/social | Engaging in hobbies | “Reading and drawing. They were helping me at the time… let me be myself in my own world… let me escape.” (CYP_012)  “Athletics… It was almost a distraction type thing… and you know, you get to meet a lot of new people, make new friends. So that was helpful as well.” (CYP_017) | “She's a very keen runner. I think, you know, having extracurricular things that the child enjoys and are good at, it doesn't just boost their confidence in that one setting, that has an overall impact on their general life, I think, and helps them to be more confident just day-to-day. So, I think that has helped and I think more things like that would only be a positive thing.” (PG_033)  “He’s very attached to [his hobby]. [He says his hobby] is so important that I don't want to do anything… he doesn’t want to do his learning.” (PG_022) |
|  | Social | Relying on social support | “I’d talk to my mom, and then I'd talk to a trusted teacher in school but they couldn't really do much to help.” (CYP_020)  “My dad is more aware, he’s sort of felt the same way as I do at the minute at some point in his life, so he's relatable to me and he suggests things that could help.” (CYP_002) | “Talking to other people, that helps a lot. Yeah, knowing that you’re not on your own. It's not only your child going through it.” (PG_027)  “It’s really hard as a parent 'cause you don't really want to talk about it with anyone because you don't want them to think you're a terrible parent because your child wants to die.” (PG_023) |
|  | Systemic | Seeking alternative services | “I saw the school counsellor and he helped me, he was just someone that I talked to and listened to the problems.” (CYP_014)    “I tried some drawing and talking therapy before… It was like a private one that my parents paid for. But it didn't really work that well, which is why I'm still with CAMHS… I was really struggling at the time, just drawing and talking wasn't really enough.” (CYP_008) | “She had a play therapist. That was really good and there were certain incidents that happened and [name of therapist] actually supported her through that. Sadly, she went and moved on to a different employment. So that then stopped.” (PG_032)  “The school counselling isn't helpful because she has it and then she literally has to go to a lesson straight after. And, you know, she's a bit, she's a bit away with the fairies and can't concentrate on the lesson. So, it has affected her studies, trying to have the counselling at school.” (PG_034) |
